# Immunomodulatory Effect of Irreversible Electroporation Alone and Its Cooperating With Immunotherapy in Pancreatic Cancer

**DOI:** 10.3389/fonc.2021.712042

**Published:** 2021-09-10

**Authors:** Guo Tian, Jiajia Guan, Yanhua Chu, Qiyu Zhao, Tian’an Jiang

**Affiliations:** ^1^Department of Ultrasound Medicine, The First Affiliated Hospital, Zhejiang University School of Medicine, Hangzhou, China; ^2^Department of Biomedicine, Key Laboratory of Pulsed Power Translational Medicine of Zhejiang Province, Hangzhou, China

**Keywords:** irreversible electroporation, immunity, pancreatic cancer, immunotherapy, ablation

## Abstract

Emerging studies have showed irreversible electroporation (IRE) focused on pancreatic cancer (PC). However, the effects of IRE treatment on the immune response of PC remain unknown. Moreover, there are few studies on the therapeutic effect of IRE combining with immunotherapy on PC. Thus, we review recent advances in our understanding of IRE alone and its working with immunotherapy towards the immune response of PC, discussing potential opportunities for exploring future treatment strategies.

## Introduction

Pancreatic cancer (PC) is one of the most lethal diseases, which has a rapid progression and a poor prognosis. The 5-year overall survival rate for PC patients is nearly 3% (1). About 80% of patients lose the chance of surgery due to tumor metastasis or local progression when PC is diagnosed (2). The adjuvant treatment of pancreatic cancer mainly includes chemoradiotherapy and local ablation. Chemoradiotherapy as a local treatment option for PC has many side effects. The traditional local ablation including radiofrequency ablation (RFA), microwave ablation (MWA), and cryoablation have significant curative effects in the solid organ tumors such as liver, kidney, and breast. However, due to the thermal damage, heat sink effects, and high risky region, their application in the treatment of pancreatic cancer is limited (3). Irreversible electroporation (IRE) is a newly developed non-thermal ablation technique for the treatment of tumors, which could induce tumor cell death along with permanent membrane lysis or loss of homeostasis by generating an extremely high electric field across cells (4–6). In particular, it has advantages for lesions located in large vessels, the hilar region, bile duct, and ureter (7). Our center previously successfully treated the lesions located in the liver (8), portal vein tumor thrombus (9), and retroperitoneum (10). The follow-up of the patient after IRE showed that the lesions were completely ablated. In addition, other center studies showed that after IRE treatment for the lesions located in the liver (11, 12), pancreas (13, 14), kidney (15, 16), and prostate (17, 18), the results show that the treatment was effective. The advantage of IRE over traditional ablation is to achieve complete ablation while reducing damage of the surrounding vessel, duct system, and peripheral nerves.

In recent years, emerging studies have evaluated immunomodulatory effect of IRE alone and its working with immunotherapy in PC. We searched PubMed, EMBASE, Web of Science, Scopus, Chinese National Knowledge Infrastructure (CNKI), Wanfang data, EBSCO, and Cochrane Library up to April 2021 for eligible studies using wide search terms: pulsed electric field, irreversible electroporation, IRE, nanoknife, nanosecond, nano-pulse, and pancreas. The screened publications were appraised by two individuals. Other literature was assessed from references in review papers. It seemed that IRE may be an important approach inducing the inflammatory immune response and host defense mechanisms. However, there are few studies on the relationship between IRE and PC immunity, and it has not been fully elucidated (19–21). Therefore, the present study aims to explore the molecular mechanisms of IRE in PC, focusing on the illustrating immunomodulatory effect of IRE alone and its cooperating with immunotherapy in PC.

## Signaling Pathway

Studies of IRE against PC have brought a greater understanding of their immunological and molecular mechanisms. Following IRE for PC, signaling downstream of epithelial growth factor receptor (EGFR) and K-RAS was decreased. AKT (22), Janus kinase (JAK), nuclear factor kappa B (NF-κB), vascular endothelial growth factor (VEGF), and signal transducers and activators of transcription 1/3 (STAT1/3) signaling downstream of EGFR, and mitogen-activated protein kinase kinase 1/2 (MEK1/2), c-Jun N-terminal kinase (JNK), and extracellular regulated protein kinases (ERK1/2) signaling downstream of K-RAS, were significantly decreased (23). Interestingly, in the rabbit VX-2 breast cancer model, it was found that IRE enhanced the antitumor immune response by reducing the plasma levels of soluble interleukin-2 receptor (sIL-2R) and transforming growth factor-β1 (TGF-β1) (24). Although TGF-β signaling was upstream of many vital signaling pathways in pancreatic cancer, there was no significant impact of IRE treatment on TGF-β signaling (23). Previous study showed that nanosecond pulsed electric field (nsPEF) could reduce antiapoptosis B-cell lymphocyte/leukemia-2 (Bcl-2) family proteins expressions [phosphorylated Bcl-2 protein (p-Bcl-2), Bcl-xL, and myeloid leukemia-1 (Mcl-1)] and increase proapoptosis Bcl-2 family proteins expressions (Bax, Bim, and BID). NsPEF caused apoptosis of human pancreatic carcinoma cell line (PANC-1) cells through the mitochondria intrinsic apoptosis pathway, which was induced by proportion disorder of anti- or proapoptosis Bcl-2 family proteins on the mitochondrial membrane (25). A study reported that nsPEF in PANC-1 cells might reduce NF-κB pathway proteins [inhibitor of kappaB kinase- alpha (IKK-α), IKK-β, inhibitor of NF-κB alpha (IκB-α), NF-κB p-65 and phosphorylated p65 (p-p65)] and cyclin proteins (cyclin D1 and cyclin A) expressions. It suggested that nsPEF restrained cell proliferation by restricting NF-κB signaling pathway for downregulating cyclin proteins expressions and inhibiting phase G1 of cell cycle (25). A study found that nsPEF in PANC-1 cells could reduce Wnt/β-catenin signaling pathway proteins [human Dapper1 (hDpr1), β-Catenin and c-Myc] and VEGF and matrix metalloproteinases (MMPs) family proteins (MMP1, MMP2, MMP9, MMP11, MMP12, MMP14, and MMP21) expressions at different degrees with different intensities of nsPEF. It indicated that nsPEF may inhibit invasion and metastasis of tumor cells by restricting Wnt/β-catenin signaling pathway to reduce expressions of VEGF and MMPs family proteins (25). Thus, the effect of IRE on KRAS, EGFR, mitochondria intrinsic apoptosis, NF-κB, and Wnt/β-catenin signaling may provide important treatment strategies. These pathways are often dysregulated in PC patients.

## The Biological Activity of IRE Alone Against PC

**Figure 1:**
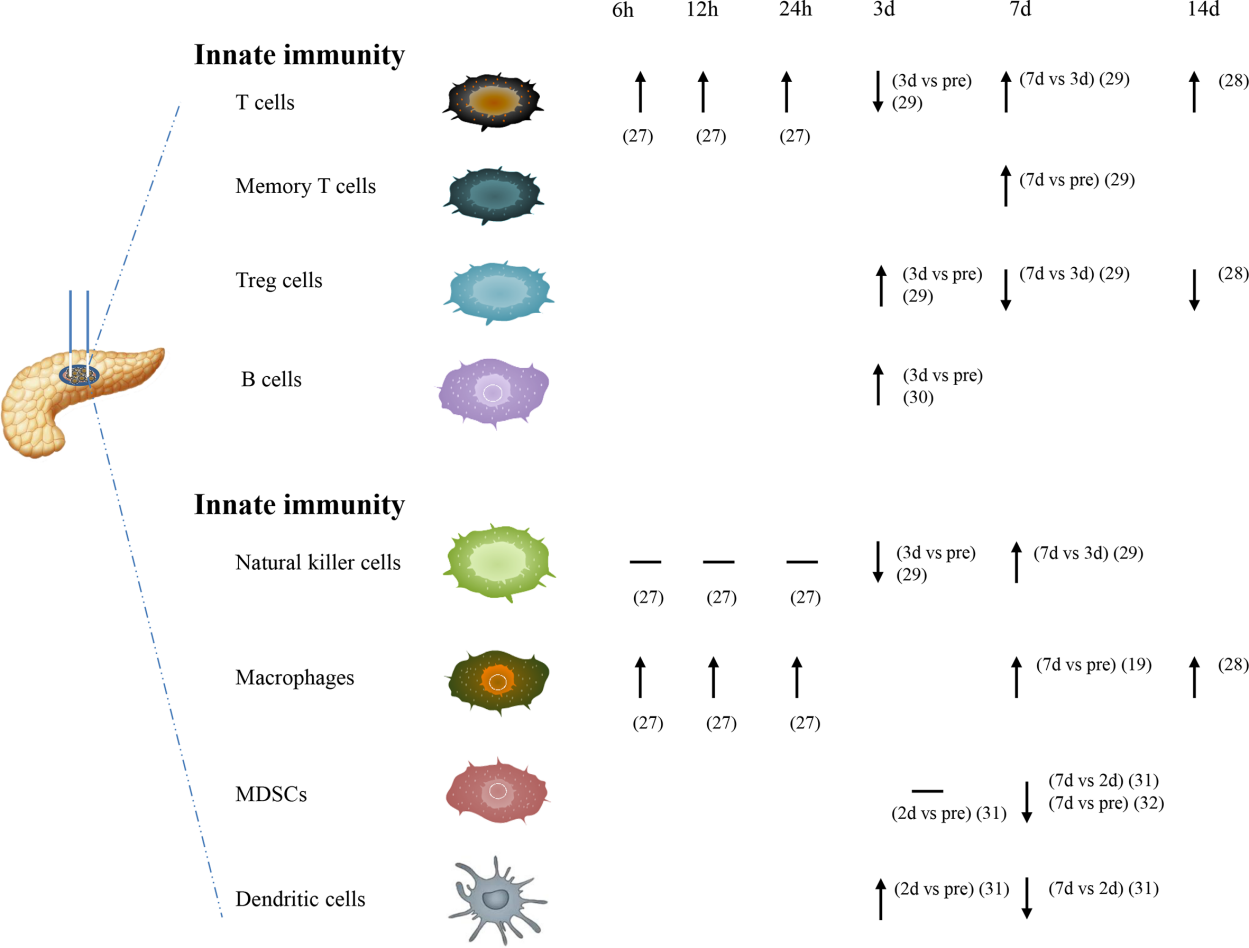
The effects of nsPEFs alone against PC on both innate and adaptive immunity.

IRE, as a potent trigger in immune responses, has direct effects on both innate and adaptive immunity (**Figure 1**). IRE could induce apoptosis in the early stages and decrease immune-suppressive cells. IRE may enhance immunotherapy efficacy because it causes a transformation from the inherently immunosuppressive microenvironment to another that was proinflammatory and antitumorigenic (26). For cancer hallmarks, cellular injury and regeneration were mainly affected by treatment. Before IRE treatment, cellular injury signaling was increased in the patient-derived xenograft (PDX) tumors but reduced after the therapy. It showed an increase in regeneration and repaired signaling with increased IRE dosage (23).

At the 6-, 12-, and 24-h time point, the number of macrophages and T cells could be more significantly increased in the IRE group. But for natural killer (NK) cells, no significant differences were found in the IRE group at these three time points. NK cells seemed to have a downward trend compared to before surgery within 24 h after IRE (27). Macrophage cells in the tumor showed a significant increase on day 7 after IRE (19). Memory T cells were also increased significantly on day 7 after IRE in tumor and lymph node (20). The significantly higher number of macrophages and T cells are detected on day 14 after IRE (28). However, a transient decrease in regulatory T cells (Treg) occurred on day 14 after the IRE procedure (21). It was reported that CD4+ T cell, CD8+ T cell, NK cell, IL-2, C3, C4, and IgG have a transitory decrease on day 3 after IRE, then a steady increase on day 7 after IRE, but Treg cell, IL-6, and IL10 have a reverse trend (29). Unlike these findings, in a total of 92 local advanced pancreatic carcinoma (LAPC) patients using IRE alone, on day 3 after treatment, the total T cell count, CD4+ T cell count, CD8+ T cell count, NK cell count, and B-cell count were obviously raised (30). Myeloid-derived suppressor cells (MDSCs) have the same level on day 2 posttreatment compared with that on pretreatment, but they were significantly reduced on day 7 posttreatment (31). Similar to another study by Jayanth Shankara Narayanan et al., it was found that MDSCs were obviously reduced on day 7 after nsPEF (32). Dendritic cells (DCs) have a transitory increase on day 2 posttreatment compare with that on pretreatment, but then were significantly reduced on day 7 posttreatment (31).

## The Immune Effect of IRE in the Treatment of PC on Spleen

In orthotopic nude-mouse models, the transformation rate of splenic lymphocytes in the IRE group was higher than that in the control group (p < 0.05). IRE may enhance the activity of splenic B lymphocytes, stimulate the body’s cellular immune function, and achieve the effect of inhibiting tumor cells (33). In a syngeneic mouse with Pan02 pancreatic cancer, it seemed that Treg cells increased to a peak in the tumor microenvironment (TME) and in the spleen 2 days after IRE treatment (31). It was reported that there was a decreased Treg cell infiltration in the spleen on day 7 and a slightly increased macrophage cell infiltration in the spleen on day 7 (19). Memory CD8+ T cells both in the spleen and lymph node increased significantly after IRE. Furthermore, the ratios of effector CD8+ T cells elevated obviously in the spleen and lymph node with the increasing strength of electroporation (20). These showed that not only in the tumor or tumor-draining lymph nodes but also in spleen that IRE increased the systematic infiltration of immune-activated cells. Further analysis was needed to check whether IRE could have similar immunomodulatory function in other organs.

## The Effect of IRE Combined With Immunotherapy on PC

**Figure 2:**
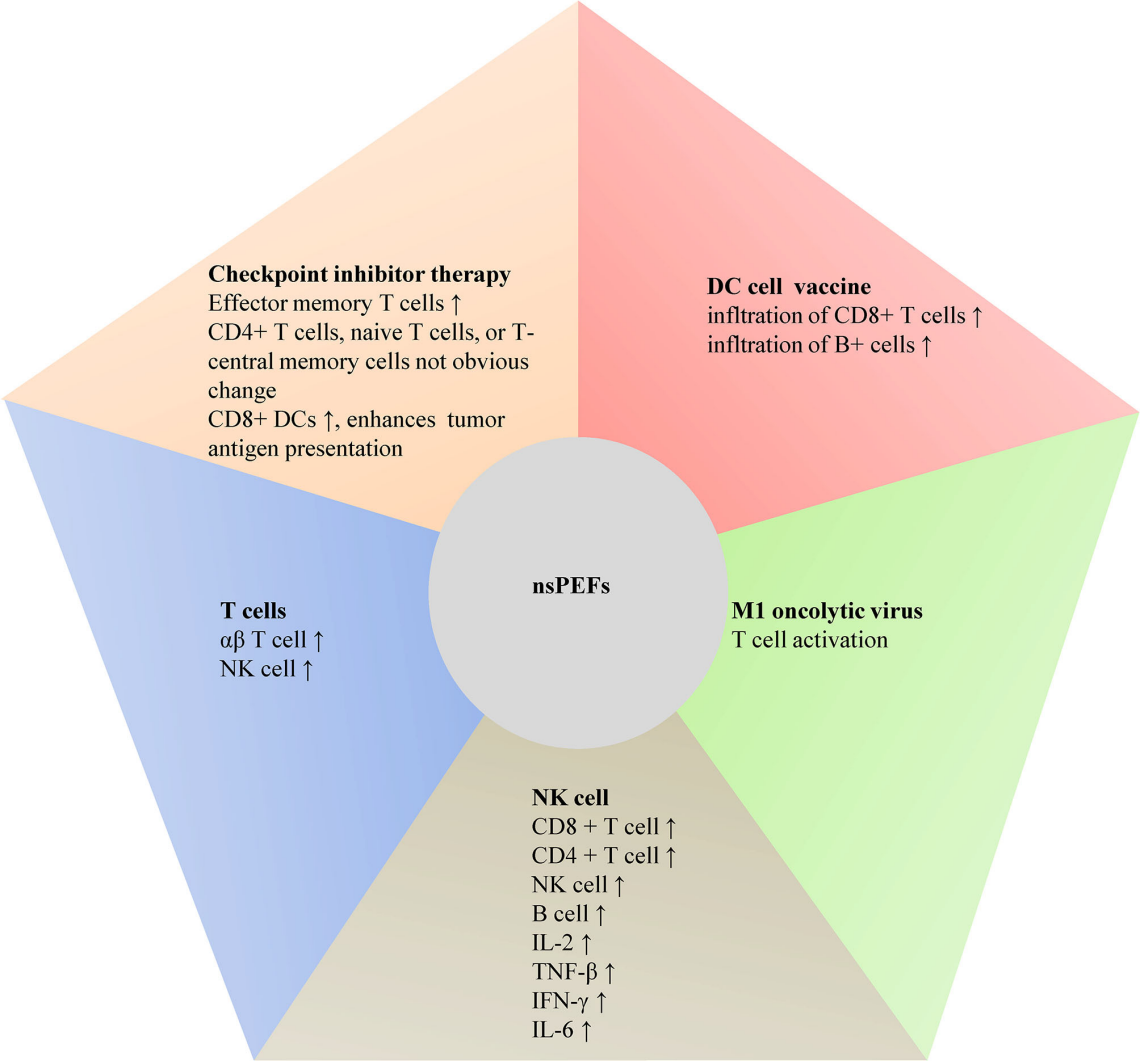
The effect of nsPEFs combined immunotherapy on PC.

It is urgent to have novel therapies and techniques to prolong the survival of PC. Pancreatic cancer lacks response to many individually applied immunotherapy (34). In recent years, electrochemotherapy (ECT), the first application of electroporation in oncology, could temporarily enhance membrane permeability *via* reversible electroporation to accelerate the transportation of bleomycin or cisplatin into tumor cells, then increase their cytotoxicity (7). ECT has been established as an efficient way for the treatment of cutaneous tumors (35). For PC, ECT seemed be promising, but it was still unclear due to the small number of studies (36–38). ECT could induce systemic antitumor T-cell responses (7). However, it may be that the antitumor immune responses raised by ECT alone were not strong enough to kill fully established distant tumors (39, 40). It seemed that the combination of ECT with immunotherapy, such as immune checkpoint inhibitors or strategies based on electrogenetherapy, could be an efficient approach for the ECT-treated lesions and distant lesions (40). Similarly, some studies showed that the immune effects of IRE alone are inadequate to clear all distant micrometastatic disease in PC patients (32, 41, 42). In some centers, they indicated that local control rates could be >90% after IRE, but many patients experienced distant recurrence (41, 43, 44). Thus, it is necessary to focus on better methods to treat micrometastasis. IRE caused by heterogeneous electric field magnitude could result in inadequate ablation and tumor recurrence. Focusing on the chemoresistance of the tumor microenvironment and the resistance of pancreatic cancer to therapy with immune checkpoint inhibitors, many efforts have been tried to improve systemic therapeutic efficacy (45). Therefore, we elaborated immunomodulatory effect of IRE cooperating with immunotherapy in PC in the following paragraphs (**Figure 2**).

## IRE Combined With Checkpoint Inhibitor Therapy on PC

Emerging studies showed that PC could not make response to immune checkpoint blockade due to limited neo-antigen expression and a poor local immunological tumor microenvironment (46). Although combination regimens including chemotherapy indicated initial promise, most combination regimens did not show favorable prognosis in survival compared with standard of care (47). Interestingly, O’Neill et al. indicated that combination therapy of IRE and programmed death ligand-1 (PD-L1) expression in murine models of pancreatic cancer was well tolerated. Effector memory T cells were increased by 1.96 times on day 90 posttreatment. There were no obvious changes among CD4^+^ T cells, naive T cells, or T-central memory cells (45). During this phase 1b clinical trial of 10 cases with stage III PC, IRE was performed followed by nivolumab. The result showed that mean time to progression was 6.3 months with median overall survival (OS) of 18.0 months. One patient had nivolumab-related adverse event, and seven patients underwent grade 3/4 treatment-related adverse events (45) (**Table 1**). Combining IRE with intratumoral toll-like receptor-7 (TLR7) agonist (1V270) and systemic antiprogrammed death-1 receptor (PD)-1 checkpoint blockade caused more than fourfold interferon (IFN)-secreting CD8+ T cells than IRE alone (32). This combination elevated the M1/M2 ratio, sand CD8+ DCs, which enhanced tumor antigen presentation to the CD8+ T cells. Survival in this mice model was significantly prolonged. Furthermore, when the cooperation improved the local effects of IRE, they could cause therapeutic abscopal effects against small secondary tumors, providing the potential for the eradication of distant micrometastatic disease (32). However, further studies are still required to identify these possibilities.

**Table 1:** Summary of IRE combined with immunotherapy on PC in human clinical studies.

Study	Study period	Objects	Intervention	After IRE Treatment	Sample size (M/F)	Age (year)	Outcome	Reference
O’Neill C et al.	July 2017–April 2018	Patients with stage III PC	IRE + checkpoint inhibitor (nivolumab)	Patients received the first dose of 240 mg intravenous nivolumab between postoperative days 1 to 5. Subsequent doses were given every 2 weeks for a total of 4 doses.	10 (4/6)	62 (38–67)	Mean time to progression was 6.3 months with median overall survival of 18.0 months. One patient had nivolumab-related adverse event; 7 patients developed grade 3/4 treatment-related adverse events.	(45)
Mao Lin et al.	June 2017–June 2018	Patients with LAPC	IRE + Vγ9Vδ2 T cell	Patients received one to three courses of Vγ9Vδ2 T-cell infusion; one course was a 28-day period, containing two infusion cycle.	30 (17/13)	63 (21–79)	Median OS: 14.5 months; patients treated with multiple courses of γδ T-cell infusion had longer OS (17 months) than those who received a single course (13.5 months).	(48)
Pan Q et al.	January 2016–January 2017	Patients with stage III PC	IRE + NK cell	Each patient was reinfused with about 300 ml of cells (1 × 10^10^ cells in total) within 3 days	46 (24/22)	56.8 ± 10.9	DFS: 7.2 ± 4.3 months; OS: 12.4 ± 5.2 months; at 1 month posttreatment, CR: 30.4%, PR: 41.3%, SD: 26.1%, PD: 2.2%, RR: 71.7%	(30)
Mao Lin et al.,	March 2016–December 2016	Patients with stage III/IV PC	IRE + NK cell	At 7 days before IRE, peripheral bloods were collected. IRE was carried out on days 9 and 12, the NK cell were infused intravenously on days 13–15.	20 (12/8)	57	At 2 months posttreatment, CR: 30.0%, PR: 50.0%, SD: 20.0%, PD: 2.2%, RR: 80.0%. The QOL was higher at both 1 month and 2 months posttreatment.	(49)
Mao Lin et al.,	March 2016–February 2017	Patients with stage III/IV PC	IRE + NK cell	At 7 days before IRE, peripheral bloods were collected. IRE was carried out on days 9 and 12, the NK cell were infused intravenously on days 13–15.	37	NA	Stage III: CR: 26.3%, PR: 36.8%, SD: 15.8%, PD: 21.1%, RR: 63.2%; median PFS: 9.1 months; median OS: 13.6 months. Median PFS and OS receiving multiple NK were higher than those receiving single NK (9.9 vs. 8.2 months, and 13.7 vs. 12.1 months). Stage IV: CR: 0, PR: 27.8%, SD: 38.9%, PD: 33.3%, RR: 26.3%; median PFS: 5.3 months; median OS: 10.2 months. Median PFS and OS receiving multiple NK were a little more than those receiving single NK (5.5 vs.5.1 months, and 10.4 vs. 9.3 months).	(50)

QOL, quality of life; PC, pancreatic cancer; IRE, irreversible electroporation; NK, natural killer; PFS, progression-free survival; OS, overall survival; CR, complete response; PR, partial response; SD, stable disease; PD, progressive disease; RR, response rate; NA, not available.

## IRE Combined With T Cell on PC

The clinical responses of immune checkpoint inhibitors (ICI) for pancreatic cancer were poor. The efficacy of ICIs in PC was prone to immunosuppressive stroma (26). The immunosuppressive microenvironment could restrain the activity of tumor infiltrating lymphocytes (51). Recently, immunotherapy has been used for tumor therapy. In the TME, T cells played an important role, and treatment of ICIs or adoptive cell infusion was promising in cancer therapy (52). Targeting both αβ T cells (CD4+, CD8+ T cells) and γδ T cells was vital in cancer immunity, which has similar features like cytotoxic effector functions and proinflammatory cytokine secretion. However, their dependence on major histocompatibility complex (MHC) molecules varied. γδ T cells consist of 0.5%–16% of CD3+ cells in the peripheral blood, which may be activated by MHC (53). In recent years, the Vγ9Vδ2 T cells have been applied against many types of cancers (54, 55). Previous study showed that IRE and allogenic Vγ9Vδ2 T cells could enhance antitumor effect for PC patients. In addition, there was significant elevation about the αβ T cell and NK cell levels after allogenic Vγ9Vδ2 T-cell infusion, and more infusion courses induced more immune cells. The median OS of LAPC patients receiving IRE and allogenic Vγ9Vδ2 T cells was 14.5 months. These patients receiving IRE plus multiple Vγ9Vδ2 T cells have longer OS (17 months) than those who received IRE plus a single course (13.5 months) (48).

## IRE Combined With NK Cell on PC

NK cells are a vital member of the innate immune system against cancers (56). *In vitro* amplification and reinfusion of NK cells indicated satisfactory prognosis in the solid malignancies treatment of the kidney (57) and breast (58). In patients with stage III PC, Pan et al. found that IRE combined with NK cells had a synergistic impact on strengthening the immune function and could decrease CA19-9 level. In IRE-NK group, it showed that at 1 month posttreatment, the rates of complete response (CR), partial response (PR), stable disease (SD), progressive disease (PD), and response rate (RR) in 46 patients were 30.4%, 41.3%, 26.1%, 2.2%, and 71.7%, respectively. The mean disease-free survival (DFS) and OS in this group reached 7.2 ± 4.3 months and 12.4 ± 5.2 months. No severe complications during IRE for PC were observed in patients intraoperatively and postoperatively (30) (**Table 1**). NK cells could identify and break down cells like tumor cells without MHC class I molecules by activating receptors (59). It seemed that more activating killer cell immunoglobulin-like receptors (KIRs) could lead to more NK activation and caused a greater antitumor effect (60). Some studies were focusing on allograft NK cells rather than autologous NK cells for immunotherapy of tumors. In the study of patients with stage III/IV PC, Lin et al. showed that IRE plus allogeneic NK cell therapy had a synergistic effect. Some lymphocyte subsets (CD8 + T cell, CD4 + T cell, NK cell, and B cell) levels and cytokine [IL-2, tumor necrosis factor beta (TNF-β), IFN-γ, and IL-6] levels were significantly higher after the treatment, which might enhance the immune function and reduce CA19-9 and CA242 level. In the IRE-NK group of 20 PC cases, a 2-month follow-up posttreatment indicated 6 cases of CR (30.0%), 10 cases of PR (50.0%), 4 cases of SD (20.0%), 0 case of PD (0%), and 16 cases of RR (80.0%). The quality of life (QOL) was better at both 1 and 2 months posttreatment (**Table 1**). Moreover, the combined IRE and NK cell treatment was well-tolerated, and the incidences of adverse reactions in the IRE-NK group were low (49), which was similar to the results of another study conducted by this team (50) (**Table 1**). These supported this combination in a promising way.

## IRE Combined With DC Vaccine on PC

Immunotherapy clears cancer cells by reducing patient tolerance to tumor-associated antigens and triggering endogenous antitumor immunity. DC therapy was a powerful immunotherapeutic method (61). However, DC immunotherapy showed limited improved prognosis in PC patients because of the immunosuppressive TME, which limited the infiltration and function of T cells. DC vaccines were obtained by culturing *ex vivo* DCs that were from patients with a specific antigen. Following maturation and activation, DCs were injected back into the patient. It showed that therapy was promising with the most common side effects like fatigue and/or flu-like symptoms. MHC class I tetramer analysis before and after vaccination indicated effective generation of antigen-specific T cells in three PC patients with stable disease (62). Yang et al. reported that IRE would overcome tumor microenvironment immunosuppression to improve the efficacy of DC cell vaccine in a mice model of PC. Their combination may cause immunogenic cell death and relieve immunosuppressive components in PC microenvironment, including increased tumor infiltration of CD8+ T cells and B+ cells (63), which well indicated that this combination exerted a synergistic effect to enhance the therapeutic efficacy of patients.

## IRE Combined With M1 Oncolytic Virus on PC

Oncolytic virotherapy meant that oncolytic viruses selectively disrupted tumor tissues by directly lysing cells, causing systemic antitumor immunity, or regulating tumor vasculature (64). M1 virus could kill residual cancer cells following IRE. Electroporation caused by IRE could offer a non-receptor-dependent membrane channel for M1 virus. IRE may regulate the tumor stroma by elevating microvessel density and tumor vessel permeability. This combined treatment could show more local concentration of M1 virus (26). The combination of IRE and M1 oncolytic virus turned immune-silent tumors into immune-inflamed tumors characterized through T-cell activation, which obviously prolonged the survival of orthotopic PC-bearing immunocompetent mice (22).

## Conclusion

In conclusion, studies of IRE on PC immunotherapy indicated new strategies by which IRE could enhance antitumor immune responses. IRE alone has direct effects on both innate and adaptive immunity in PC. IRE cooperating with immunotherapy may play an important role in further prolonging survival of PC patients. However, many questions were still urgent about the properties and functions of IRE in PC. For example, less is known about how to measure the metabolic switch of immune cells during IRE in PC, which is an essential issue for understanding immunometabolic regulations in immune cells. The exciting area of immuno-oncology could be meaningful for prolonging the survival of PC patients. Large-scale prospective randomized controlled trials will be necessary to identify these findings, thus offering references for the options of treatment protocols for PC patients.

## Author Contributions

Study concept and design: GT. Acquisition of data: GT, JG, YC, QZ, and TJ. Analysis and interpretation of data: GT and QZ. Drafting of the manuscript: GT. Critical revision of the manuscript for important intellectual content: TJ. Statistical analysis: JG and YC. Obtaining of funding: TJ and QZ. Technical or material support: TJ. Study supervision: TJ. All authors contributed to the article and approved the submitted version.

## Funding

This study was supported by the Development Project of National Major Scientific Research Instrument (82027803), National Natural Science Foundation of China (81971623 and 82171937), Key Project of Natural Science Foundation of Zhejiang Province (LZ20H180001), and Zhejiang Provincial Association Project for Mathematical Medicine (LSY19H180015).

## Conflict of Interest

The authors declare that the research was conducted in the absence of any commercial or financial relationships that could be construed as a potential conflict of interest.

## Publisher’s Note

All claims expressed in this article are solely those of the authors and do not necessarily represent those of their affiliated organizations, or those of the publisher, the editors and the reviewers. Any product that may be evaluated in this article, or claim that may be made by its manufacturer, is not guaranteed or endorsed by the publisher.
